# The ROX index (Index combining the respiratory rate with oxygenation) is a prognostic factor for acute respiratory distress syndrome

**DOI:** 10.1371/journal.pone.0282241

**Published:** 2023-02-27

**Authors:** Kenta Nishiyama, Kazuya Ichikado, Keisuke Anan, Kazunori Nakamura, Kodai Kawamura, Moritaka Suga, Takuro Sakagami

**Affiliations:** 1 Division of Respiratory Medicine, Saiseikai Kumamoto Hospital, Kumamoto, Japan; 2 Department of Respiratory Medicine, Faculty of Life Sciences, Kumamoto University, Kumamoto, Japan; Bilawal Medical College, Liaquat University of Medical and Health Sciences, PAKISTAN

## Abstract

**Background:**

There is no existing reliable and practical method for predicting the prognosis of acute respiratory distress syndrome (ARDS).

**Objective:**

We aimed to clarify the association between the ROX index, which is calculated as the ratio of peripheral oxygen saturation divided by the fraction of inspired oxygen to the respiratory rate, and the prognosis of patients with ARDS under ventilator support.

**Methods:**

In this single-center retrospective cohort study from prospectively collected database, eligible patients were categorized into three groups based on ROX tertiles. The primary outcome was the 28-day survival, and the secondary outcome was 28-day liberation from ventilator support. We performed multivariable analysis using the Cox proportional hazards model.

**Results:**

Among 93 eligible patients, 24 (26%) patients died. The patients were divided into three groups according to the ROX index (< 7.4, 7.4–11, ≥ 11), with 13, 7, and 4 patients dying in the groups, respectively. A higher ROX index was associated with lower mortality; adjusted hazard ratios [95% CIs] for increasing tertiles of ROX index: 1[reference], 0.54[0.21–1.41], 0.23[0.074–0.72] (P = 0.011 for trend) and a higher rate of successful 28-day liberation from ventilator support; adjusted hazard ratios [95% CIs] for increasing tertiles of ROX index: 1[reference], 1.41[0.68–2.94], 2.80[1.42–5.52] (P = 0.001 for trend).

**Conclusions:**

The ROX index at 24 h after initiating ventilator support is a predictor of outcomes in patients with ARDS and might inform initiation of more advanced treatments.

## Introduction

Acute respiratory distress syndrome (ARDS) is a potentially fatal condition induced by various pulmonary or extrapulmonary diseases that can cause non-cardiogenic pulmonary edema. The global burden of ARDS remains high, with approximately three million patients being diagnosed with ARDS annually. ARDS accounts for approximately 10% of intensive care unit admissions. Moreover, 24% of patients under mechanical ventilation in the intensive care unit suffer from ARDS [1, 2]. The severity and prognosis of ARDS should be considered during treatment decision-making.

The severity of ARDS is widely classified using the Berlin definition. This categorization system also includes treatment decisions, such as the initiation of extracorporeal membrane oxygenation, and prognosis assessment [3]. Moreover, it was validated in a patient-level meta-analysis of 4188 patients with ARDS, which found a hospital mortality rate of 27%, 32%, and 45% for mild, moderate, and severe ARDS, respectively [4]. However, there is controversy surrounding the use of the Berlin definition. Specifically, it is limited by inconsistencies in the time of measurement of the arterial oxygen tension (PaO_2_)/fraction of inspired oxygen (FiO_2_) ratio after ventilator support initiation [5] and variations in the PaO_2_/FiO_2_ ratio based on the positive end-expiratory pressure (PEEP) [6] and tidal volume [7]. Furthermore, the Berlin definition has been considered as unreliable for predicting prognosis [8]. There is a need to develop new measures that consider ventilator settings, including PEEP. Recently, the ratio of peripheral oxygen saturation (SpO_2_) divided by the FiO_2_ to the respiratory rate (ROX index) has been determined as a predictor of positive outcomes after high-flow nasal cannula therapy in patients with severe pneumonia [9, 10].

However, the association of the ROX index with prognosis in patients with ARDS under ventilator support remains unclear. We aimed to clarify the association of the ROX index with the prognosis of patients with ARDS under ventilator support. We hypothesized that a low ROX index is associated with poor prognosis.

## Materials and methods

This study was performed according to the Strengthening the Reporting of Observational Studies in Epidemiology statement [11] (S1 Table).

### Study design and patient selection

This single-center retrospective observational study was conducted following the tenets of the Declaration of Helsinki and was approved by the Institutional Ethics Committee of the hospital (No: 898). The requirement of informed consent was waived given the retrospective study design. We selected patients from the database [12] of Saiseikai Kumamoto Hospital, which serves as an advanced emergency hospital. This database includes patients diagnosed with ARDS based on the American-European Consensus Conference on ARDS [13] from October 2004 to June 2012, and based on the Berlin definition of ARDS [4] from July 2012 to July 2019. All data, including those in the database, were originally collected continuously and prospectively in our previous study [14–17]. From this database, we selected patients who received ventilator support using an endotracheal tube or non-invasive positive pressure ventilation through a mask for ARDS treatment from October 2011 to July 2019. We excluded patients who died within 24 h of initiating ventilator support, as well as those treated with neuromuscular blockers, high-flow nasal cannula therapy, or extracorporeal membrane oxygenation. Moreover, we excluded patients with pre-existing chronic interstitial lung diseases or other interstitial lung diseases, including acute organizing pneumonia, hypersensitivity pneumonitis, and acute eosinophilic pneumonia. Patients who had underlying cardiogenic edema or Human Immunodeficiency Virus were included, unless they had any exclusion criterion.

### The ROX index

The ROX index was defined as the ratio of SpO_2_ divided by FiO_2_ (%) to the respiratory rate (breaths/min) [9, 10]. From the records, we noted the patients’ respiratory rate 24 h after ventilator support initiation, and the ROX index was calculated at 24 h after ventilator support initiation. For patients without records at 24 h after ventilator support initiation, we noted the respiratory rate within 2 hours of scheduled timing.

### Outcomes

The primary outcome was the 28-day mortality, and the secondary outcome was successful 28-day liberation from ventilator support. The patients were followed up until death or the 28^th^ day in the hospital. Survival was indicated as being alive on the 28^th^ day of hospitalization.

### Data collection

Demographic variables were recorded at the time of ARDS diagnosis. Within 24 h of admission to the intensive care unit, we calculated the intensive care unit Acute Physiology and Chronic Health Evaluation (APACHE) II score [18], which is the classification system of disease severity calculated using initial values of 12 routine physiologic measurements, age, and previous health status to provide a general measure of severity of disease, and the Sequential Organ Failure Assessment [19] scores, which is a measure of the degree of organ function failure calculated using six domains involving the respiratory, cardiovascular, hepatic, coagulation, renal, and neurological systems. Moreover, the etiology of ARDS, with respect to whether there was direct or indirect lung injury, was recorded [20]. We assessed findings from chest radiography and blood tests performed before treatment. Additionally, the occurrence of organ failure before and during ventilator support was recorded. Age and PaO_2_/FiO_2_ ratio at ARDS diagnosis were considered as confounding variables.

### Ventilator support

Ventilator strategies included lung-protective ventilation and optimal PEEP with the following ARDS management objectives [21]: to control tidal volume at 6–8 mL/kg of the ideal body weight (not exceeding 10 mL/kg) and to achieve optimal PEEP. The parameters for each device were set by the physician. Multiple respiratory specialists reached a consensus regarding liberation from mechanical ventilation.

### Sample size

Since ARDS is a rare condition, we collected as many patients as possible without calculating the sample size.

### Statistical analyses

There were no missing data regarding the ROX index and confounders; therefore, we performed a complete case analysis. Continuous and categorical variables are presented as median (range) and number (percentage), respectively. Patients were categorized into three groups based on the ROX tertiles: low (< 7.4), intermediate (7.4–11), and high (≥ 11). Survival curves were described using the Kaplan–Meier method. We performed univariate and multivariate analyses using Cox proportional hazard models to compare the hazard ratios for each group with those of the reference (low ROX) group. In the multivariate analysis, we adjusted for age and the PaO_2_/FiO_2_ ratio. Moreover, we performed the log-rank trend test using the median ROX value within each tertile as an ordinal value. Statistical significance was set at p < .05. Statistical analyses were performed using EZR software (Saitama Medical Center, Jichi Medical University, Saitama, Japan) [22] and STATA/SE ver. 16.0 (Stata Corp., College Station, TX, USA).

## Results

### Patient characteristics

**Fig 1:**
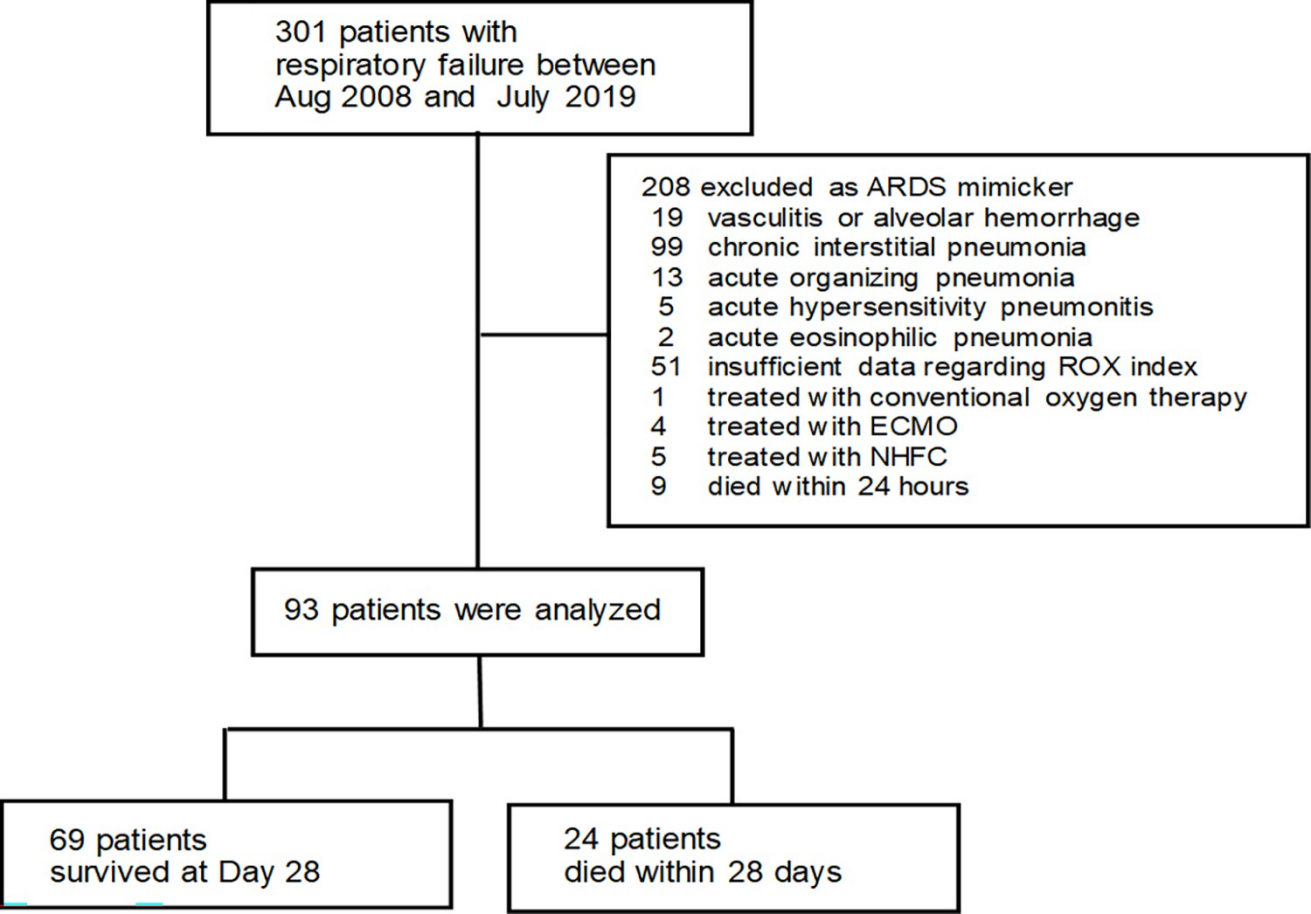
A flowchart showing patient inclusion, exclusion, and outcomes. ECMO: extracorporeal membrane oxygenation, ARDS: acute respiratory distress syndrome, NHFC: nasal high-flow cannula.

Fig 1 presents the patient flowchart. Among 425 eligible patients, 332 patients were excluded for various reasons, with the remaining 93 patients being included in the analysis. Table 1 presents the demographic characteristics and baseline clinical characteristics. Among the included patients, 68 (73%) patients required endotracheal intubation, while 49 (53%) patients were diagnosed with direct lung injury. Sixty-eight patients (73%) had ARDS from infection. Compared with the other groups, the low ROX group showed a lower PaO_2_/FiO_2_ ratio for ARDS diagnosis, rate of endotracheal intubation, C-reactive protein levels, and PEEP, as well as a higher age and APACHE II scores on admission. In the low, intermediate, and high ROX groups, 13, 7, and 4 patients died, respectively; moreover, 13, 16, and 25 patients were successfully liberated from ventilator support within 28 days, respectively.

**Table 1:** Baseline characteristics of the enrolled patients.

Variables	Total (n = 93)	ROX index < 7.4 (n = 31)	7.4 ≤ ROX index < 11 (n = 30)	ROX index ≥ 11 (n = 32)
Age, years	73 [19–97]	77 [36–91]	69 [44–87]	74 [19–97]
Sex, male (%)	62 (67)	19 (61)	19 (63)	24 (75)
Lung injury etiology Direct, n (%)	47 (51)	17 (55)	15 (50)	15 (47)
Pneumonia, n (%)	39 (42)	13 (42)	15 (50)	11 (34)
Aspiration, n (%)	8 (9)	4 (13)	0 (0)	4 (13)
Indirect, n (%)	34 (37)	10 (32)	10 (33)	14 (44)
Non-pulmonary sepsis, n (%)	20 (22)	8 (26)	5 (17)	7 (22)
Drug, n (%)	10 (11)	1 (3)	4 (13)	5 (16)
Others, n (%)	4 (4)	1 (3)	1 (3)	2 (6)
Unknown, n (%)	12 (13)	4 (13)	5 (17)	3 (9)
PaO_2_/FiO_2_ (mmHg)	139 [50–300]	110 [50–260]	140 [53–300]	160 [57–280]
White blood cell count (/μL)	9500 [300–43000]	6000 [300–43000]	12000 [300–25000]	12000 [1100–30000]
C-reactive protein (mg/L)	15 [0.03–53]	14 [1.8–47]	15 [0.03–53]	17 [0.43–44]
Lactate dehydrogenase (U/L)	320 [120–2300]	300 [150–1500]	440 [120–2300]	300 [180–1100]
Platelet count (×10^4^/μL)	16 [2.0–42]	14 [1.9–27]	16 [2.6–32]	18 [5.1–42]
Serum albumin (g/dL)	2.7 [1.4–4.1]	2.7 [1.4–4.1]	2.7 [1.5–3.6]	2.7 [1.4–3.7]
Positive end-expiratory pressure (cmH_2_O)	9.0 [5.0–28]	8.0 [5.0–22]	9.0 [5.0–20]	10[5.0–28]
Missing, n (%)	1(1.1)	0	1(3.3)	0
Peak inspiratory pressure (cmH_2_O)	21 [6.0–30]	19 [10–27]	22 [6.0–28]	20 [8.0–30]
Missing, n (%)	31 (33)	11 (35)	10 (33)	10 (31)
Tidal volume (mL)	420 [250–720]	420 [250–720]	420 [300–650]	430[300–670]
Missing, n (%)	30 (32)	11 (35)	9 (30)	10 (31)
Ventilation type, endotracheal intubation, n (%)	68 (73)	17 (55)	26 (87)	25 (78)
Prone position	0 (0)	0 (0)	0 (0)	0 (0)
Severity of ARDS, mild/ moderate/ severe	17 (18)/44 (47)/32 (34)	3 (9.7)/17 (55)/11 (35)	7 (23)/13 (43)/10 (33)	7 (22)/14 (44)/11 (34)
APACHE II score	20 [10–36]	22 [11–36]	21 [12–32]	19 [10–29]
SOFA score	8.0 [2.0–17]	8.0 [3.0–16]	9.0 [2.0–17]	7.0 [2.0–15]
McCabe classification category, 1/2/3	71 (76)/15 (16)/7 (7.5)	21 (68)/7 (23)/3 (9.7)	23 (77)/5 (17)/2 (6.7)	27 (84)/3 (9.3)/2 (6.3)

Continuous and categorical variables are presented as median [range] and the number of patients (percentage), respectively.

APACHE, Acute Physiology and Chronic Health Evaluation; ARDS, acute respiratory distress syndrome; PaO_2_/FiO_2_, arterial oxygen tension divided by the fraction of inspired oxygen; SOFA, Sequential Organ Failure Assessment.

### Twenty-eight-day mortality

**Fig 2:**
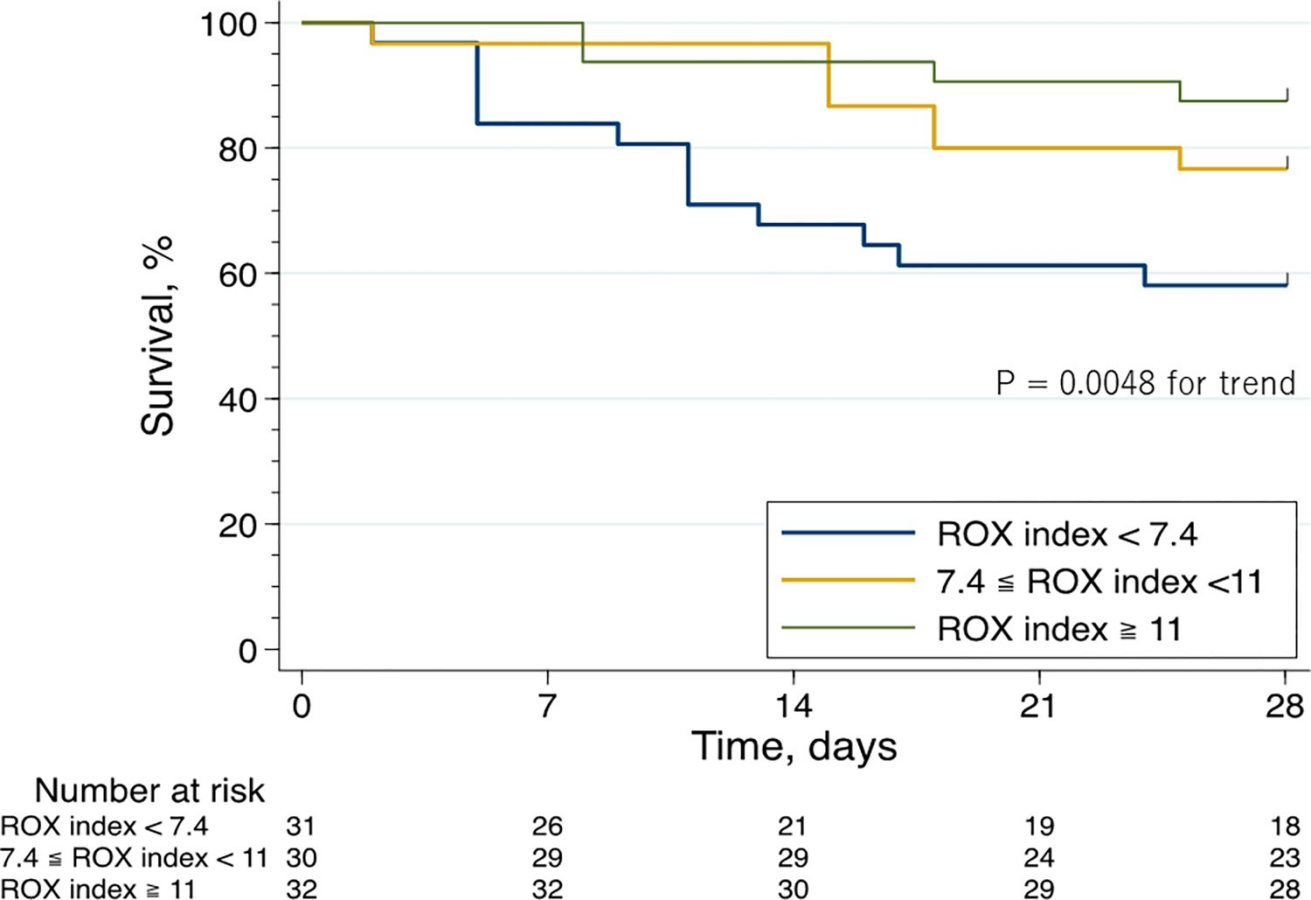
Kaplan–Meier curve analysis of the 28-day survival stratified by the ROX index.

The Kaplan–Meier curve and log-rank trend test revealed an inverse relationship between the ROX index and mortality (Fig 2). Similarly, univariate and multivariate analyses (Table 2) showed that a higher ROX index was associated with lower mortality (adjusted hazard ratios [95% confidence intervals (CIs)] for increasing tertiles of ROX index: 1 [reference], 0.54 [0.21–1.41], 0.23 [0.074–0.72]; P = 0.011 for trend).

**Table 2:** Correlation of the ROX index with outcomes.

	28 day mortality			
	ROX index <7.4	7.4≦ROX index <11	ROX index ≧ 11	P for trend
Number of patients	31	30	32	
Number of events	13	7	4	
Univariate analysis, HR (95% CI)	1[reference]	0.45 (0.18, 1.14)	0.24 (0.077, 0.72)	0.0048
Multivariate analysis*, HR (95% CI)	1[reference]	0.54 (0.21, 1.41) *	0.23 (0.074, 0.72) *	0.011
** **	**Ventilator liberation by day 28**			
Number of events	13	16	25	
Univariate analysis, HR (95% CI)	1[reference]	1.41 (0.68, 2.93)	2.81 (1.43, 5.51)	0.0013
Multivariate analysis*, HR (95% CI)	1[reference]	1.41 (0.68, 2.94)*	2.80 (1.42, 5.52)*	0.001

The ROX index was defined as the ratio of peripheral oxygen saturation divided by the fraction of inspired oxygen (%) to the respiratory rate (breaths/min). *Adjusted for age and PaO_2_/FiO_2_ ratio

HR, hazard ratio; CI, Confidence Interval.

### Successful liberation from ventilator support by the 28^th^ day

**Fig 3:**
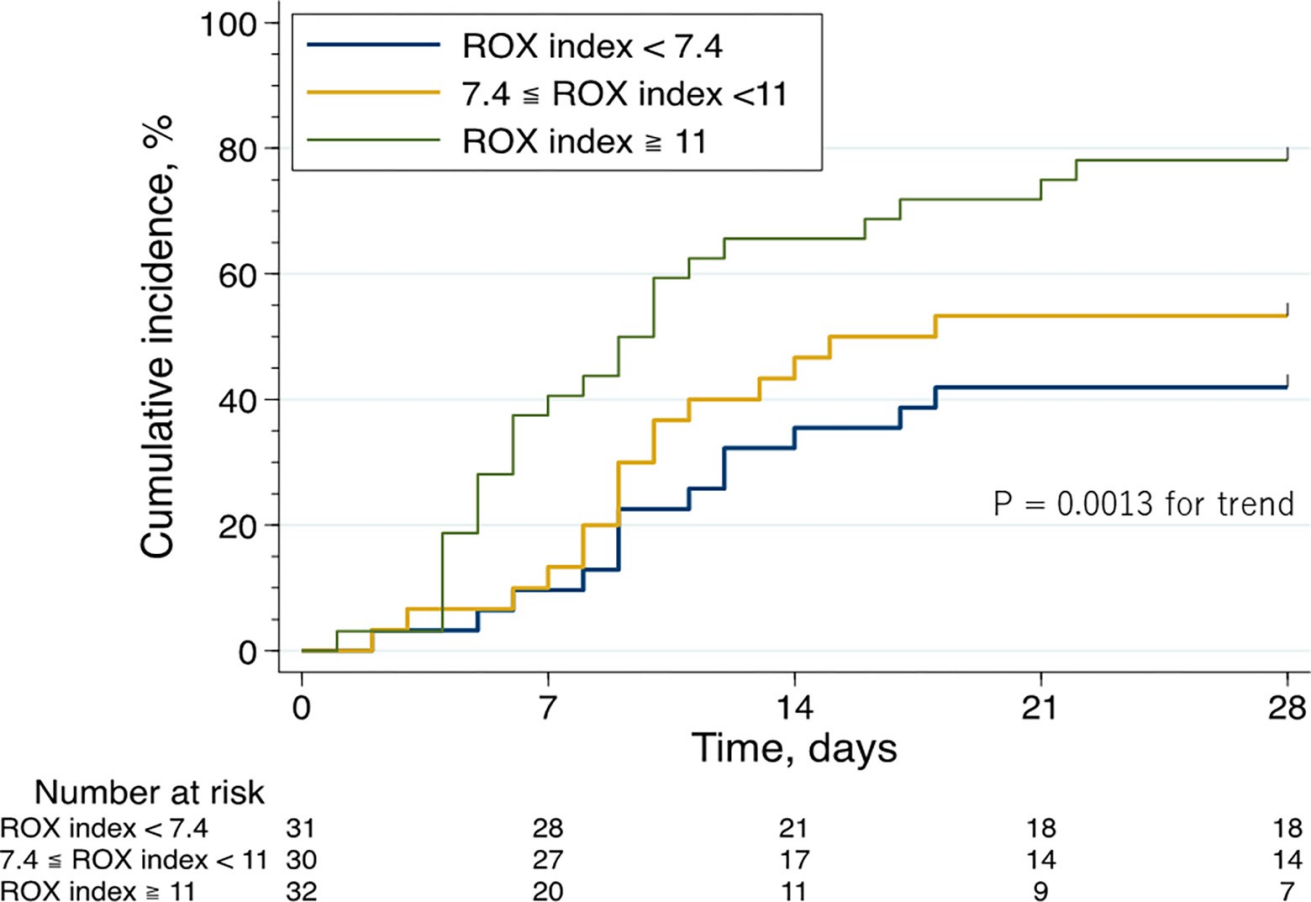
Kaplan–Meier curve analysis for the 28-day ventilator liberation stratified by the ROX index.

The Kaplan–Meier curve and log-rank trend test revealed that the ROX index was directly associated with the rate of successful liberation from ventilator support by 28 days (Fig 3). Similarly, univariate and multivariate analyses (Table 2) showed that a higher ROX index was associated with a higher rate of successful liberation from ventilator support by the 28^th^ day (adjusted hazard ratios [95% CIs] for increasing tertiles of ROX index: 1 [reference], 1.41 [0.68–2.94], 2.8 [1.42–5.52]; P = 0.001 for trend).

## Discussion

This study investigated the association between the ROX index and ARDS prognosis. To our knowledge, this is the first study to investigate the association of the ROX index in patients with ARDS under ventilator support, except for high-flow nasal cannula therapy, with prognosis. We found that the ROX index was associated with outcomes. Moreover, the ROX index was inversely and directly associated with the 28-day mortality rate and rate of ventilator withdrawal, respectively.

Our findings demonstrate the usefulness of the ROX index in patients with ARDS under ventilator management. A high ROX index has been reported to predict positive outcomes, including successful liberation from high-flow nasal cannula therapy and low intubation rates [9, 10]. However, these previous studies included patients undergoing high-flow nasal cannula therapy, while we included patients with ventilator support. We found that a higher ROX index was associated with better outcomes, including low mortality and ventilator weaning by day 28, which is consistent with previous findings.

The ROX index is a convenient bedside measure for assessing prognosis. ARDS is currently classified based on the Berlin definition; however, there is still controversy regarding this. Specifically, severity measurement is limited by inconsistencies in the timing of the measurement of the PaO_2_/FiO_2_ ratio after ventilator support initiation [5] and variations in the PaO_2_/FiO_2_ ratio according to PEEP [6] and tidal volume [7]. Moreover, several biomarkers associated with ARDS have been identified [23]. Studies on biomarkers have further elucidated the pathogenesis of ARDS and indicated the involvement of fibrinolysis [24] and acute inflammation [25, 26], as well as various cells such as lung epithelial cells [27, 28] and endothelial cells [29, 30]. However, there is no single biomarker that can be solely used to predict prognosis. Although combining several biomarkers [31] or fibroproliferative changes on high-resolution computed tomography images [12] can be used to predict prognosis, difficulties in bedside measurements of these data limit their clinical utility. We found that ROX is associated with the prognosis of ARDS after adjustment for age and the PaO_2_/FiO_2_ ratio at diagnosis; therefore, it could be a novel measure for assessing the prognosis of ARDS.

Our findings demonstrated that a lower ROX index was associated with poor prognosis. Hypoxia, high FiO_2_, and tachypnea lead to a low ROX index. Moreover, non-invasive ventilation and low PEEP are closely associated with hypoxia and tachypnea, which lead to a low ROX index. This indicates that the ROX index might reflect the severity of respiratory failure, general conditions such as acidemia, and labored breathing. In the low ROX group, there were more elderly people, patients without endotracheal intubation, and patients with low PEEP. Even after adjustment for age and the PaO_2_/FiO_2_ ratio, the ROX index was associated with poor outcomes. This suggests that the ROX index might reflect both the respiratory status and the severity of general conditions, as well as insufficient analgesia and sedation.

### Clinical implications

The ROX index may be useful as an indicator for considering advanced treatments. A low ROX index was associated with poor outcomes adjusted for age and the PaO_2_/FiO_2_ ratio. It could be considered as a measure of the severity of general condition, analgesia deficiency, sedation, PEEP, and pressure support. Therefore, patients with a low ROX index should be considered for more advanced treatments, including intubation, prone position, neuromuscular blockades, and extracorporeal membrane oxygenation. Since the ROX index can be easily calculated at the bedside, it can inform prompt treatment decisions.

### Research implication

The appropriate timing and the cut-off value of the ROX index remain unclear. We calculated the ROX index 24 h after ventilator support initiation based on a previous report that risk stratification based on the Berlin definition was improved by assessing the PaO_2_/FiO_2_ ratio 24 h after enrollment rather than at baseline [32]. Further studies are required to clarify the timing and cut-off value.

### Strengths and limitations

This is the first study to investigate the association of the ROX index with outcomes in patients with ARDS receiving ventilator support. This study has several limitations. First, this was a single-center retrospective study with a small sample size using a prospectively collected dataset. Compared with a typical retrospective design, our study is strengthened by the use of a prospectively enrolled cohort including prospective identification of acute respiratory failure as suspected ARDS. However, it may introduce an unintentional bias in patient selection. It is unclear whether our findings are useful in other settings; therefore, there is a need for multicenter prospective studies. Second, we only included patients with ARDS; therefore, our findings cannot be generalized to all patients with acute respiratory failure.

## Conclusions

Our findings demonstrated that the ROX index measured 24 h after ventilator support initiation was a predictor of outcomes in patients with ARDS. The ROX index can be easily assessed at the bedside and may be useful for informing prompt advanced treatments.

## Supporting information

S1 Table STROBE statement—checklist of items that should be included in reports of observational studies. (DOCX)
