# Supplementary material for: The ROX index (Index combining the respiratory rate with oxygenation) is a prognostic factor for acute respiratory distress syndrome
Source: PLoS One. 2023 Feb 27;18(2):e0282241. doi: 10.1371/journal.pone.0282241 (PMC9970069; doi:10.1371/journal.pone.0282241)
Supplement: S1 Table — (DOCX) [file pone.0282241.s001.docx]

**S1 Table.** **STROBE Statement—checklist of items that should be included in reports of observational studies.**

|  | **Item number** | **Recommendation** | **Reported on Page** |
| --- | --- | --- | --- |
| Title and Abstract | 1 | (a) Indicate the study’s design with a commonly used term in the abstract | 3 |
|  |  | (b) Provide in the abstract an informative and balanced summary of what was found | 3 |
| **Introduction** |  |  |  |
| Background | 2 | Explain the scientific background and rationale for the investigation being reported | 4 |
| /rationale Objectives | 3 | State specific objectives, including any prespecified hypotheses | 5 |
| **Methods** |  |  |  |
| study design | 4 | Present key elements of study design early in the paper | 5 |
| setting | 5 | Describe the setting, locations, and relevant dates, including periods of recruitment,  exposure, follow-up, and data collection | 5, 6 |
| Participants | 6 | Cohort study—Give the eligibility criteria, and the sources and methods of selection of  participants. Describe methods of follow-up | 6 |
| variables | 7 | Clearly define all outcomes, exposures, predictors, potential confounders, and  effect modifiers. Give diagnostic criteria, if applicable | 6, 7 |
| Data sources | 8 | For each variable of interest, give sources of data and details of methods of assessment  (measurement). | 7 |
| / measurement |  | Describe comparability of assessment methods if there is more than one group | None |
| Bias | 9 | Describe any efforts to address potential sources of bias | 8 |
| study size | 10 | Explain how the study size was arrived at | 8 |
| Quantitative variables | 11 | Explain how quantitative variables were handled in the analyses. If applicable, describe  which groupings were chosen, and why | 8 |
| Statistical methods | 12 | (a) Describe all statistical methods, including those used to control for confounding | 8 |
|  |  | (b) Describe any methods used to examine subgroups and interactions | None |
|  |  | (c) Explain how missing data were addressed | None |
|  |  | (d)Cohort study—If applicable, explain how loss to follow-up was addressed | None |
|  |  | (e) Describe any sensitivity analyses | None |
| **Results** |  |  |  |
| Participants | 13 | (a) Report the numbers of individuals at each stage of the study—e.g., numbers potentially eligible, examined for eligibility, con-firmed eligible, included in the study, completing follow-up, and analysed | 9 |
|  |  | (b) Give reasons for non-participation at each stage | Figure 1 |
|  |  | (c) Consider use of a flow diagram | Figure 1 |
| Descriptive data | 14 | (a) Give characteristics of study participants (e.g., demographic, clinical, social) and  information on exposures and potential con-founders | 9, 10 |
|  |  | (b) Indicate the number of participants with missing data for each variable of interest | 10, 11 |
|  |  | (c)Cohort study—Summarise follow-up time (e.g., average and total amount) | 9 |
| Outcome data | 15 | Cohort study—Report numbers of outcome events or summary measures over time | 13 |
| Main results | 16 | (a) Give unadjusted estimates and, if applicable, confounder-adjusted estimates and their precision (e.g., 95% confidence interval). Make clear which confounders were adjusted for and why they were included | 13 |
|  |  | (b) Report category boundaries when continuous variables were categorized | 9 |
|  |  | (c) If relevant, consider translating estimates of relative risk into absolute risk for  a meaningful time period | None |
| Other analyses | 17 | Report other analyses done—e.g., analyses of subgroups and interactions,  and sensitivity analyses | None |
| **Discussion** |  |  |  |
| key results | 18 | Summarise key results with reference to study objectives | 15 |
| Limitations | 19 | Discuss limitations of the study, taking into account sources of potential bias or  imprecision. Discuss both direction and magnitude of any potential bias | 18 |
| Interpretation | 20 | Give a cautious overall interpretation of results considering objectives, limitations,  multiplicity of analyses, results from similar studies, and other relevant evidence | 17 |
| Generalisability | 21 | Discuss the generalisability (external validity) of the study results | 18 |
| **Other information** |  |  |  |
| funding | 22 | Give the source of funding and the role of the funders for the present study and,  if applicable, for the original study on which the present article is based | 2 |
